# Oral intermittent vitamin D substitution: influence of pharmaceutical form and dosage frequency on medication adherence: a randomized clinical trial

**DOI:** 10.1186/s40360-020-00430-5

**Published:** 2020-07-11

**Authors:** Jean-Pierre Rothen, Jonas Rutishauser, Philipp N. Walter, Kurt E. Hersberger, Isabelle Arnet

**Affiliations:** 1 Pharmaceutical Care Research Group, Department of Pharmaceutical Sciences, University of Basel, Petersplatz 14, Postfach 2148, CH-4001 Basel, Switzerland; 2 Nutrimed Ltd, Basel, Switzerland; 3 Division of Endocrinology, Diabetes, and Metabolism, University Hospital, Basel, Switzerland; 4 Solothurn Hospitals, Institute for Laboratory Medicine, Olten, Switzerland

**Keywords:** Cholecalciferol, Oral intermittent treatment, Adherence, Preference, Formulation, Dosage frequency

## Abstract

**Background:**

To assess adherence to and preference for vitamin D substitution with different pharmaceutical forms and frequencies of administration.

**Methods:**

A focus group of stakeholders aimed at preparing the design of an interventional, randomized, cross-over study with 2 × 2 groups obtaining monthly or weekly vitamin D products in liquid or solid form for 3 months each. Dosage corresponds to cumulated amount of recommended 800 IU daily (5.600 IU weekly / 24.000 IU monthly). Main inclusion criteria were a vitamin D serum value < 50 nmol/l and age ≥ 18 years. Primary endpoint was adherence, secondary endpoints were preferences and vitamin D serum levels.

**Results:**

The focus group reached consensus for preference of a monthly administration of solid forms to adults.

Full datasets were obtained from 97 participants. Adherence was significantly higher with monthly (79.5–100.0%) than weekly (66.4–98.1%) administration. Vitamin D levels increased significantly (*p* < 0.001) in all participants. An optimal value of > 75 nmol/l was achieved by 32% after 3 months and by 50% after 6 months. Preferred formulation was solid form (tablets, capsules) for 71% of participants, and preferred dosage frequency was monthly for 39% of participants.

**Conclusions:**

Monthly oral vitamin D in solid form lead to the highest adherence, and is preferred by the participants. However, only one third of study participants achieved values in the optimal range of > 75 nmol/l cholecalciferol using weekly or monthly administration providing an average daily cholecalciferol dose of 800 IU.

**Trial registration:**

NCT03121593 | SNCTP000002251. Registered 30. May 2017,. Prospectively registered.

## Background

An adequate supply of vitamin D can hardly be achieved with a usual diet and under normal exposition to sun light in temperate latitudes. Vitamin D deficiency occurs frequently, especially during winter months [1, 2] and year around in elderly people who can only produce a reduced amount of vitamin D in their skin [3]. Other risk factors include dark skin (since melanin impairs the effect of UV-B radiation), overweight (because vitamin D is trapped in fatty tissue), lack of exercise, and cultural or religious dress codes (due to underexposure of the skin to sunlight) [4–7]. Hypovitaminosis D appears to play an important role in a number of extraskeletal diseases such as several autoimmune diseases [8, 9] or Alzheimer’s and Parkinson’s disease [10]. Vitamin D plays a key role in various physiological processes such as the regulation of brain development and activities in adulthood [11]. The serum value is considered the most significant indicator for vitamin D storage. Serum levels < 50 nmol/l indicate a deficiency and are associated in adults with secondary hyperparathyroidism, osteomalacia or osteoporosis, proximal limb muscle weakness, ataxia, and increased risk of falls, increased risk of fractures, or hampered effect of drugs used for osteoporosis [12]. Values < 25 nmol/l indicate a severe deficiency. Optimum values > 75 nmol/l are recommended in subjects with osteopenia, osteoporosis or fragility fractures, and in patients on treatment for osteoporosis [12], without exact definitions of the upper reference value [13]. Vitamin D can be supplemented in deficient individuals at every age for therapeutic or prophylactic purposes. However, recommendations differ. The US Institute of Medicine recommends a daily intake of 600 IU for adults aged 19–59, 800 IU for those aged > 60 and 1.500–2.000 IU vitamin D for those with severe vitamin D deficiency [14]. The maximum tolerable amount is 4.000 IU vitamin D per day [14]. The upper limit for adults according to the Endocrine Society Clinical Practice Guideline is 10.000 IU vitamin D per day [8].

Due to a vitamin D half-life of about 2 months [15], intermittent weekly or monthly intake of cumulative doses of cholecalciferol achieves the same serum values as daily intake [16–18].

Recommended cumulative doses and frequencies of intermittent administration vary widely: 300.000 IU annually [19], 50.000 IU twice weekly [13, 20] or 20–25.000 IU weekly [16, 21]. Compared with 24.000 IU vitamin D monthly, the administration of 60.000 IU monthly or additional 24.000 IU calcifediol resulted in 25-hydroxyvitamin D levels above 75 nmol/l more often. However, the improvement of the lower extremity function did not differ among the treatment groups, while the incidence of falls differed significantly, with higher incidences in the 60.000 IU group and the 24.000 IU plus calcifediol group [22].

Pharmaceutical formulation can impact patient adherence [23]. In addition, the intake is more reliable with intermittent administration [24, 25]. In Switzerland, several oily and alcoholic solutions are currently commercially available for oral daily or intermittent use as well as newly formulated tablets for daily use.

This study aimed to explore which formulation and dosage frequency of vitamin D would ensure the highest adherence.

## Methods

### Focus group

A focus group is a moderated discussion with the aim of finding a consensus among experts on a specific issue [26]. Our focus group consisted of 10 individuals experienced with vitamin D administration, who are 5 healthcare professionals (2 family doctors, 2 community pharmacists, 1 home care nurse) and 5 patients. They rated the preferred pharmaceutical dosage form and dosage frequency separately for infants, adults and seniors on a yes / no / indifferent answer scale. Consensus was defined as unanimous, strong (≥90% identical votes) or firm (≥70% identical votes). Importance of reimbursement and physician’s knowledge were answered with a 5-point Likert scale (from − 2: not important to + 2: very important). Open discussion was facilitated in case of discordant voting before re-voting.

### Intervention study

**Fig. 1:**
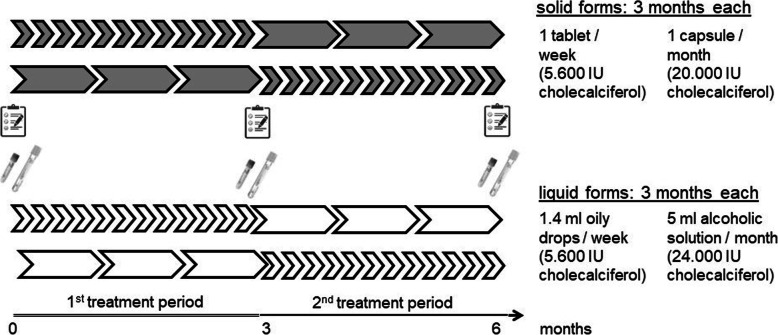
Study design: Patients obtained vitamin D either as tablets (5.600 IU) and capsules (20.000 IU) or 1.4 ml oily drops (5.600 IU) and 5 ml alcoholic drinking solution (24.000 IU), for weekly and monthly administration over 3 months, respectively. Blood samples  and questionnaires  were obtained at inclusion, 3 and 6 months

We performed an interventional, randomized, 2 × 2 groups cross-over study with administration of monthly or weekly vitamin D supplements in two liquid or two solid formulations for 6 months during winter time (Fig. 1). Study medications in liquid formulation were the 5 ml bottle Vi-De 3® monthly dose (24.000 IU/5 ml alcoholic solution) and the 30 ml bottle including a graduated pipette Vitamin D_3_ Streuli® weekly dose (5.600 IU/1.4 ml oily solution). Solid medications consisted of the capsule Dekristol® monthly dose (20.000 IU) and the tablet Dekristolvit® weekly dose (5.600 IU). Each participant was randomized to a solid or liquid group with cross-over to the other solid or liquid medication after 3 months. All strengths and dosages frequencies correspond to cumulative doses delivering on average the recommended 800 IU for daily intake for adults, except for capsules. Solid medication of vitamin D containing 24′000 IU were not commercially available. Participants were enrolled in 7 general practices in Basel. Informed consent was obtained from all participants. Primary endpoint was adherence (taking and timing), secondary endpoints were preferences and increase of vitamin D serum levels.

Main inclusion criteria were ambulatory patients ≥18 years, vitamin D serum value < 50 nmol/l at baseline and at least one prescribed oral medication. Main exclusion criteria were hypercalcaemia and nephrolithiasis. Randomisation was achieved by removing a sealed envelope from a box containing 8 CRF (case report form) for weekly or monthly administration in random order after 4 × 4 block randomization.

Adherence was assessed electronically. We reblistered solid forms into disposable punch cards with an electronic foil affixed on the back side [27]. For liquid forms, a Time4Med™ smart card [28] was handed out to participants who registered the intake by pushing the button. A time stamp (date and time) was generated for each removal of a tablet or a capsule from the punch card (for solid forms), or for pressing the button on the device (for liquid forms).

We calculated two adherence values from the time stamp series. Taking adherence expresses the percentage of doses taken and is calculated as [doses taken / doses prescribed] × 100. Timing adherence expresses the percentage of doses taken within a preset time window of ±7.5%, that is in an allowed interval of 6.475–7.525 days for weekly schedule, and of 27.75–32.25 days for monthly schedule. We performed a visual inspection of the returned bottles.

Serum levels of 25(OH) vitamin D (reference range 50–250 nmol/l), parathyroid hormone (reference range 1.59–12.0 pmol/l), calcium (reference range 2.10–2.55 mmol/l), phosphorus (reference range 0.74–1.52 mmol/l), alkaline phosphatase (reference range 40–150 U/l), magnesium (reference range 0.66–1.07 mmol/l), creatinine were measured at the Institute for Laboratory Medicine of the Solothurn hospitals using the Architect analysis system from Abbott GmbH & Co, complete blood count using the Symex XN-Series from Sysmex Inc., at baseline, 3 and 6 months.

Three questions were asked about the intake of the specific product and one question about unexpected events during the prior 3 months: 1) How did you manage to take your medication in the past 3 months? (very good, good, bad, very bad); 2) How do you rate the pharmaceutical form of the past 3 months? (very pleasant, pleasant, unpleasant, very unpleasant); 3) How do you rate the frequency of [monthly / weekly] intake? (too frequent, ideal, too rare); 4) Have you noticed any unexpected events? (Yes (please specify), No). For evaluation of the preference, all answers were dichotomized in positive and negative statements.

During the final follow-up, general preferences were also asked: 5) Do you prefer a vitamin D preparation in solid or liquid form? (necessarily liquid, rather liquid, doesn’t matter, rather solid, necessarily solid); 6) Which intake frequency is ideal for you? (daily, weekly, monthly, annually, doesn’t matter); 7) Do you prefer [tablets or capsules? (for participants with solid forms) / alcoholic or oily drops? (for participants with liquid forms)]. Additionally, the question was asked whether the dosing pipette was an asset or a drawback (appropriate answers; doesn’t matter).

Power calculation showed that 118 patients would suffice to detect a difference of 25% in timing adherence between solid and liquid forms with a 80% power. The statistical evaluation was carried out using SPSS® (IBM, version 25). The following statistical tests were used: Mann-Whitney U-test to compare different groups, Wilcoxon to compare the results of two time points, Friedman to compare three time points and Spearman to evaluate correlations. *p*-values < 0.05 were considered significant. Arithmetical mean of Likert-Scale answers was calculated.

This study was prospectively registered and approved by the local ethics committee (EKNZ-Nr. 2017–00300).

## Results

### Focus group

Participants preferred unanimously tablets or capsules for adults and seniors, in a monthly frequency for adults and daily or weekly for seniors, especially those with multicompartment adherence aids (strong consensus). For infants, i.e. those who cannot be administered solid dosage forms, a weekly administration was unanimously preferred by the participants, with oily drops (strong consensus).

Reimbursement by health insurance was considered of moderate importance (mean + 0.4, range –from − 2 to + 2). Physicians wanted to know if their patients were taking vitamin D (mean + 1.5, range –from 0 to + 2), even if they were not the prescribers, particularly in elderly people, for instance in cases of renal insufficiency or hypercalcaemia. This attitude was understandable for the other participants of the focus group.

### Intervention study

A total of 106 patients were enrolled between October 2017 and April 2018, i.e. winter months in Basel. Nine patients dropped out due to protocol violations, including failure to initiate treatment or missing the 3-month control visit (three patients each), and study termination due to nausea, hospitalization, or death (one patient each). Ninety-seven complete datasets were available for evaluation (Table 1). 
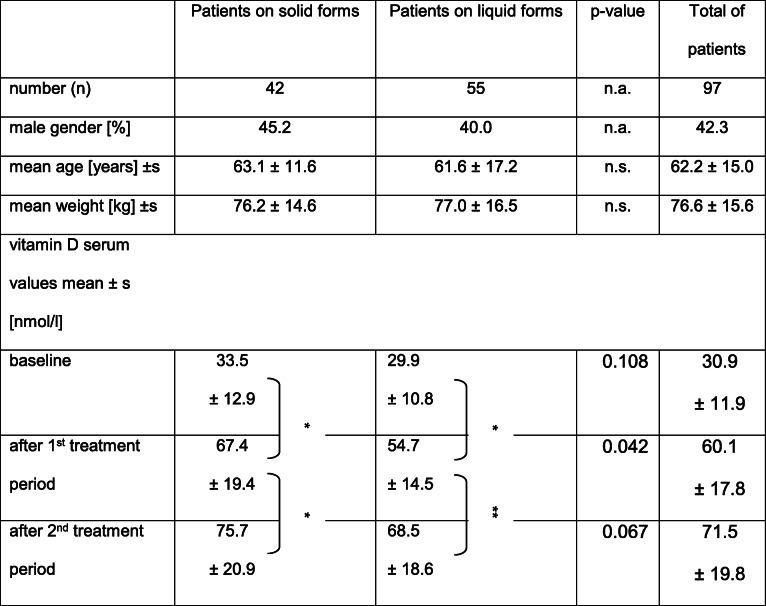

**Table 1:**
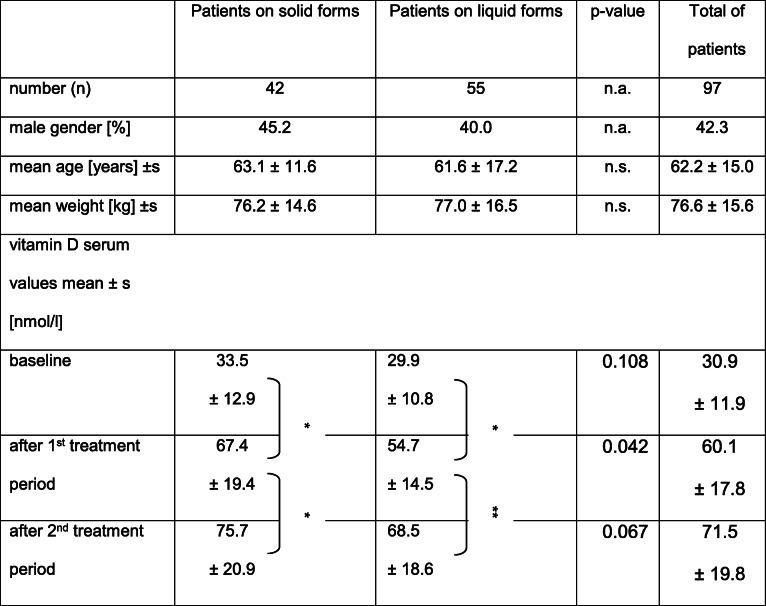
Characteristics of the study population and vitamin D serum values at baseline and after 3 months’ treatment periods. Values > 75 nmol/l are considered optimal

* *p*-value < 0.01

** *p*-value 0.01

### Adherence related to dosage frequency

Taking adherence was significantly higher during the first 3 months of treatment compared to the second 3 months, independently of the formulation (99.0% vs. 94.7%; *p* = 0.001; data not shown). Taking and timing adherence were significantly higher with monthly administration compared to weekly administration, independently of the formulation (Table 2). 
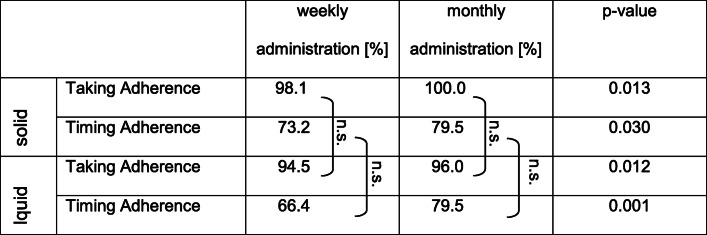

**Table 2:**
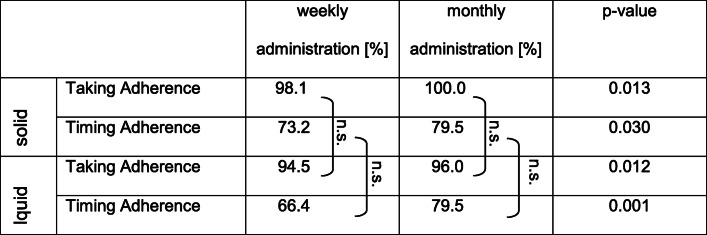
Taking and timing adherence values (average) according to pharmaceutical forms (solid and liquid) and dosage frequency (weekly and monthly). Non-significance is marked with “n.s”

### Adherence related to pharmaceutical formulation

Within a dosage frequency (weekly or monthly), the adherence did not differ significantly between the pharmaceutical formulation (Table 2). From the returned bottles with a graduated pipette, 28% contained too much or too little expected residual volume. All participants returning bottles with clearly too much residual solution achieved suboptimal serum values.

### Serum values

Patient’s baseline vitamin D values were similar in both solid and liquid preparations groups (*p* = 0.108). The vitamin D values of all participants increased significantly in both treatment periods (*p* < 0.001) and with each pharmaceutical formulation (Table 1). After two treatment periods, the values of solid and liquid formulations were similar (*p* = 0.067).

Values in the reference range > 50 nmol/l were not reached by 29% of participants after 3 months and by 9% after 6 months of treatment, respectively. Values in the optimal range of > 75 nmol/l were reached by 18% of participants after 3, and 37% of them after 6 months of treatment, respectively. No values above the reference range were observed. The highest value measured in the study was 144 nmol/l.

Values in the optimal range of > 75 nmol/l were reached by 32% of the participants with the solid dosage after the first treatment period, by 50% after the second treatment period. In the participants who were treated with drops, the proportion was 7 and 26% respectively.

### Other biomarkers

With treatment, serum parathyroid hormone levels [pmol/l] decreased significantly from 7.75 at baseline to 7.30 after the first treatment period and 7.19 after the second treatment period (*p* = 0.036). Similarly and as expected, mean serum phosphorus concentrations [mmol/l] increased under treatment from 1.51 (baseline) to 1.53 and 1.92 (*p* < 0.001), serum alkaline phosphatase [IU/l] decreased from 83 (baseline) to 81 and 76 (*p* = 0.001). Serum levels of all other biomarkers tested remained unchanged, e.g. mean serum calcium levels [mmol/l] were 2.41 (baseline), 2.40 (first treatment period) and 2.43 (second treatment period).

### Adverse events

Adverse reactions were not serious nor could they be associated with hypercalcaemia (Table 3).

**Table 3:** Listing of all reported unexpected events after 3 or 6 months of cumulative weekly or monthly oral treatment with 800 IU cholecalciferol/day (translated from Swiss German)

Preparation	Unexpected event
oily drops	fatigue, but also occurred before
alcoholic drops	possible fatigue, unclear whether there is a connection
oily drops	uncharacteristic abdominal pain
oily drops	shingles: under Vit D considerably less frequent
oily drops	less tired than usual
alcoholic drops	generally feels better under vitamin D, is better off in the morning
oily drops	foot swelling left 1 week ago - now better
oily drops	nervous, palpitations
alcoholic drops	weight loss 7 kg in 2 months (patient report not validated)
alcoholic drops	feeling, I let more water than usual (suspected diuretic effect)
alcoholic drops	heat feeling, feverish, sleep problems
alcoholic drops	had once cramp in the leg
oily drops	less pain in the hand
alcoholic drops	localized itching
alcoholic drops	a little gas (flatulence)
oily drops	possibly more diarrhea (developed over time)
oily drops	at the first 3 doses some nausea after ingestion, spontaneously gone
capsules	approx. 10 days after the 1st ingestion night sweat outbreak (one-time)
oily drops	unique feeling of palpitations

### Preferences

From the 97 participants who finished the study, all gave their preferences for each formulation taken during 3 months (tablet vs. capsule or alcoholic vs oily drops), Regarding management (question 1) coping was more often bad and very bad with weekly drops compared with weekly tablets (Chi^2^ = 5.85; *p* = 0.015). Regarding how pleasant the intake was (question 2), more participants indicated unpleasantness with liquid drops, independently of the frequencies (Chi^2^ = 18.4; *p* < 0.01). Regarding frequencies (question 3), fewer individuals estimated that monthly intake was too frequent, independently of the formulation (Chi^2^ = 17.66; *p* < 0.01).

A total of 29 patients (30%) had no preference for any galenic formulation (question 5). Of the 68 remaining participants, 71% (*n* = 48) opted for a solid form, and 29% (*n* = 20) for a liquid form (*p* < 0.001). Half of the participants with prior experience of liquid formulations changed the sides. Only one patient who had taken capsules and tablets preferred the liquid forms.

When identical efficacy was assumed for different dosage frequencies (question 6), monthly intake was favored by 36.6% of the participants, weekly intake by 19.4%, annual intake by 11.8% and daily intake by 8.6%. Frequency did not matter for 23.7% of participants.

From the participants who had taken solid dosage forms, the type of formulation would not matter for 54%; 34% preferred capsules, and 12% tablets (question 7). Of those who had taken liquid dosage forms, the type of formulation did not matter for 24%; 72% preferred drops (oily or alcoholic formulation). The dosing pipette for the oily solution was considered a drawback for 53% of participants, and an advantage for 17% of them.

## Discussion

We investigated which formulation and dosage frequency of oral vitamin D are best to promote high adherence in adult outpatients in need of a cholecalciferol substitution. We followed Swiss regulation on treatment recommendations and administered equivalents of 800 IU daily in liquid form (weekly oily drops of 5′600 IU or monthly alcoholic solution of 24′000 IU) and solid from (weekly tablet of 5′600 IU or monthly capsule of 20′000 IU). A higher adherence was observed with monthly administration, independently of the formulation. However, after 6 months of intake, a solid dosage form such as tablets or capsules was preferred together with monthly frequency. Thus, monthly administration of solid forms seems most suitable. Remarkably these results correspond to the conclusions of the focus group’s discussion prior to study initiation. After three and 6 months of intake, all serum values of vitamin D had increased. However, optimal serum values were reached with solid substitution only by half of the participants and with liquid substitution by even only 26% of them. Potential reasons for this difference may be related to inaccurate dosing using the pipette, or a larger proportion of participants under solid forms who terminated the study in spring time. An influence of sun radiation cannot be excluded. On the other hand, the strength of the capsules was lower than intended. Therefore, differences in serum values between the two formulations need careful interpretation.

Some exceptions to a monthly administration of a solid form may exist for specific groups of patients. For example, patients with a multicompartment adherence aid may prefer a daily or a weekly administration. For infants who are not able to swallow capsules or tablets, liquid dosage form as non-alcoholic product may be preferable. Weekly or daily dosing seems easiest to manage for parents.

Vitamin D values increased in all treatment groups; however, 82 and 63% of the patients 63% x did not reach an optimal value > 75 nmol/l after three and 6 months of intake, respectively. Our data clearly show that the administration of 800 cholecalciferol IU/day given as cumulative dose is not sufficient to treat profound vitamin D deficiency. These findings are in line with an earlier trial conducted with a similar number of participants [29]. A dosing scheme that reliably leads to sufficient (> 50 nmol/l) or optimal (> 75 nmol/l) vitamin D serum values without exceeding the maximum dosage of 4.000 IU vitamin D per day as indicated by the Institute of Medicine [14] would be desirable.

The advantage of a dosing pipette was not evident to most study participants. The task of measuring the correct dose was not successfully achieved in 28% of patients, despite individual instructions from the investigating doctors. To avoid this kind of error, liquid solution could be manufactured to deliver an entire dose such as a drink ampule in analogy to a capsule or a tablet.

We considered the tolerance of 24 h for weekly intake which corresponds to a grace period of 15% appropriate for an adherence study. However, the acceptance limit for timing adherence with a range of 15% is discriminatory compared to more common 25% [30]. For actual implementation outside an adherence trial, the intake interval of vitamin D is far less critical.

Our study has several strengths. First, instead of retrospective patient surveys, we used electronic devices to assess adherence. Thus, we obtained objective adherence values that are comparable over the different dosage frequencies without recall bias, which represents the main drawback of adherence questionnaires. Second, only patients with vitamin D deficiency of < 50 nmol/l were included in our study. Third, four preparations were compared which were commercially available in Switzerland or Germany. Thus, our results can be generalised and used to develop further formulations.

The study also shows some limitations. First, adherence values can be expected to be higher in volunteers who have been informed in detail of treatment objectives and are aware of monitoring procedure. Thus, the higher adherence measurement in the first treatment period could result from this Hawthorne effect. Second, the recommendation of 800 IU cholecalciferol daily corresponds to the cumulative dose of 24.000 IU per month [14]. No such solid product was commercially available in Switzerland nor in the surrounding countries (France, Germany, Italy, Austria). Thus, we used the closest dosage as possible which is 20.000 IU in form of capsules. Consequently, we can assume that the measured effect of monthly vitamin D capsules would have been even more pronounced with 24.000 IU cholecalciferol. Third, we terminated the study after the recruitment of 106 instead of 128 participants end of April. This decision was made to avoid the influence of sunlight on individual serum vitamin D values. We preferred having a smaller sample size than biased laboratory findings.

## Conclusions

An intermittent monthly oral therapy of vitamin D in solid form leads to the highest adherence, and is preferred by most adult participants. Although the cumulative dosage of 800 IU cholecalciferol per day leads to an increase of vitamin D serum values in all participants, only a minority achieved values in the optimal range > 75 nmol/l cholecalciferol. A dosing scheme that reliably leads to sufficient (> 50 nmol/l) or optimal (> 75 nmol/l) vitamin D serum values would be desirable. Physicians should investigate preferences in view of shared-decision making in the treatment choice.

## Supplementary information

**Additional file 1.** Questionnaire during the 3-months visit and the final examination: English translation. The questions were asked to the patients in German.

## Data Availability

The datasets during and/or analysed during the current study available from the corresponding author on reasonable request.
