# Supplementary material for: Oral intermittent vitamin D substitution: influence of pharmaceutical form and dosage frequency on medication adherence: a randomized clinical trial
Source: BMC Pharmacol Toxicol. 2020 Jul 11;21:51. doi: 10.1186/s40360-020-00430-5 (PMC7353738; doi:10.1186/s40360-020-00430-5)
Supplement: Supplementary file 1 — Additional file 1. Questionnaire during the 3-months visit and the final examination: English translation. The questions were asked to the patients in German. [file 40360_2020_430_MOESM1_ESM.docx]

| 3 – Months Visit |
| --- |

Remember the vitamin D preparation in the last 3 months:

| 🗣 Have you noticed any unexpected events? | | |
| --- | --- | --- |
| ⬜ | ⬜ |  |
| Yes | No |  |

If you ticked "yes": Describe the events: ____________________________ __________________

| 🗣 How did you cope with taking your medication in the last 3 months? | | | |
| --- | --- | --- | --- |
| ⬜ | ⬜ | ⬜ | ⬜ |
| very good | good | bad | very bad |

If you ticked “bad” or “very bad”: What caused you problems?

| 🗣 How did you experience the specific formulation in the last 3 months? | | | |
| --- | --- | --- | --- |
| ⬜ | ⬜ | ⬜ | ⬜ |
| very  pleasant | rather  pleasant | rather  unpleasant | very  unpleasant |

| 🗣 How do you rate the frequency of administration? | | |
| --- | --- | --- |
| ⬜ | ⬜ | ⬜ |
| too frequent | ideal | too seldom |

| Final Examination |
| --- |

Remember the vitamin D preparation in the last 3 months:

| 🗣 Have you noticed any unexpected events? | | |
| --- | --- | --- |
| ⬜ | ⬜ |  |
| Yes | No |  |

If you ticked "yes": Describe the events: ____________________________ __________________

| 🗣 How did you cope with taking your medication in the last 3 months? | | | |
| --- | --- | --- | --- |
| ⬜ | ⬜ | ⬜ | ⬜ |
| very good | good | bad | very bad |

If you ticked “bad” or “very bad”: What caused you problems?

| 🗣 How did you experience the specific formulation in the last 3 months? | | | |
| --- | --- | --- | --- |
| ⬜ | ⬜ | ⬜ | ⬜ |
| very  pleasant | rather  pleasant | rather  unpleasant | very  unpleasant |

| 🗣 How do you rate the frequency of administration? | | |
| --- | --- | --- |
| ⬜ | ⬜ | ⬜ |
| too frequent | ideal | too seldom |

Now remember the administration of the two vitamin D preparations in the past 3 months and in the 3 months before:

| 🗣 In the course of the study, i.e. in the past 6 months, were you abroad? | | |
| --- | --- | --- |
| ⬜ | ⬜ |  |
| Yes | No |  |

If you ticked "yes", in which country were you and how long?

Country:

Duration of stay:

| 🗣 If you could choose: Would you prefer a vitamin D preparation in liquid form (drops) or solid form (tablets or capsules)? | | | | |
| --- | --- | --- | --- | --- |
| ⬜ | ⬜ | ⬜ | ⬜ | ⬜ |
| definitely in liquid form | rather in  liquid form | does not matter | in solid form | definitely in solid form |

| 🗣 If the effectiveness were the same: What would be the ideal frequency of administration? | | | | |
| --- | --- | --- | --- | --- |
| ⬜ | ⬜ | ⬜ | ⬜ | ⬜ |
| once a day | once a week | does not matter | once a month | once a year |

A question only for participants who have been administered solid forms:

| 🗣 In the monthly doses you have taken capsules, in the weekly doses tablets. Which formulation do you prefer? | | |
| --- | --- | --- |
| ⬜ | ⬜ | ⬜ |
| capsules | does not matter | tablets |

Two questions only for participants who have been administered liquid forms:

| 🗣 The preparations contained alcoholic or oily drops. Apart from the fact that one preparation was available as a complete dose, the other had to be measured with a dosing pipette: Would you prefer alcoholic or oily drops? | | |
| --- | --- | --- |
| ⬜ | ⬜ | ⬜ |
| alcoholic drops | does not matter | oily drops |

| 🗣 In the weekly doses you had to use the dosing pipette. Do you consider the dosing pipette as an advantage or a disadvantage? | | |
| --- | --- | --- |
| ⬜ | ⬜ | ⬜ |
| I consider the use of the dosing pipette to as an advantage | does not matter | I consider the use of the dosing pipette as a disadvantage |
